# Enhanced Production of Biosurfactant from *Bacillus subtilis* Strain Al-Dhabi-130 under Solid-State Fermentation Using Date Molasses from Saudi Arabia for Bioremediation of Crude-Oil-Contaminated Soils

**DOI:** 10.3390/ijerph17228446

**Published:** 2020-11-15

**Authors:** Naif Abdullah Al-Dhabi, Galal Ali Esmail, Mariadhas Valan Arasu

**Affiliations:** Addiriyah Chair for Environmental Studies, Department of Botany and Microbiology, College of Science, King Saud University, P.O. Box 2455, Riyadh 11451, Saudi Arabia; gesmail@ksu.edu.sa (G.A.E.); mvalanarasu@ksu.edu.sa (M.V.A.)

**Keywords:** *Bacillus subtilis* strain Al-Dhabi-130, biosurfactant, optimization, response surface methodology, crude oil, bioremediation

## Abstract

Crude oil and its derivatives are the most important pollutants in natural environments. Bioremediation of crude oil using bacteria has emerged as a green cleanup approach in recent years. In this study, biosurfactant-producing *Bacillus subtilis* strain Al-Dhabi-130 was isolated from the marine soil sediment. This organism was cultured in solid-state fermentation using agro-residues to produce cost-effective biosurfactants for the bioremediation of crude-oil contaminated environments. Date molasses improved biosurfactant production and were used for further optimization studies. The traditional “one-variable-at-a-time approach”, “two-level full factorial designs”, and a response surface methodology were used to optimize the concentrations of date molasses and nutrient supplements for surfactant production. The optimum bioprocess conditions were 79.3% (*v*/*w*) moisture, 34 h incubation period, and 8.3% (*v*/*v*) glucose in date molasses. To validate the quadratic model, the production of biosurfactant was performed in triplicate experiments, with yields of 74 mg/g substrate. These findings support the applications of date molasses for the production of biosurfactants by *B. subtilis* strain Al-Dhabi-130. Analytical experiments revealed that the bacterial strain degraded various aromatic hydrocarbons and n-alkanes within two weeks of culture with 1% crude oil. The crude biosurfactant produced by the *B. subtilis* strain Al-Dhabi-130 desorbed 89% of applied crude oil from the soil sample. To conclude, biosurfactant-producing bacterial strains can increase emulsification of crude oil and support the degradation of crude oil.

## 1. Introduction

Petroleum hydrocarbon is an important source of energy and causes serious pollution to the environment in oil-producing countries. An uncontrolled landfill, accidental spills, leaking underground storage tanks, and improper storage lead to severe contamination to the environment [1]. Physicochemical methods are widely used in the remediation of petroleum hydrocarbon. In recent times, bioremediation of pollutants has received much more attention, because it is cost-effective, environmentally friendly, and efficient [2,3,4]. Bioremediation refers to the total conversion or elimination of toxic compounds into ecofriendly nontoxic forms using microbial consortia, bacteria, or fungi [5]. Nevertheless, the biodegradation process is critically impeded by the low solubility of petroleum hydrocarbon and poor capacity of the microbial consortium or bacterium to degrade and access the substrate. However, the bioavailability of various hydrocarbons can be increased by applying various surfactants to the contaminated sites to allow the rapid uptake of bacteria. Additionally, the use of biosurfactants in the oil field during water flooding enhances oil recovery in the oil field. Biosurfactants are produced by a range of microorganisms and are a diverse group of chemical compounds with unique physiochemical and biochemical properties. Based on their microbial origin and chemical composition, biosurfactants are classified as lipopeptides, glycolipids, polymeric type, fatty acids, and particulate biosurfactants. The most commonly reported biosurfactants are lipopeptides (rhamnolipids, surfactin and iturin, sophorolipids) and glycolipids. *Bacillus* strains are important sources for the production of lipopeptides [6]. Biosurfactants are amphiphilic compounds that have both hydrophobic and hydrophilic moieties in their chemical structure and can effectively reduce both intersurface and surface tensions and are highly useful in emulsification processes. Biosurfactants form micelles to enhance the process of hydrocarbon emulsification in the aqueous phase and critically enhance their bioavailability for microbial utilization and biodegradation [7]. Biosurfactants have good environmental compatibility, low toxicity, higher foaming activity, higher selectivity, specific activity, and better biodegradability in extreme culture conditions, such as high salinity, pH, and temperature. These properties added advantage to biosurfactants as a good alternative to costly chemically synthesized surfactant to degrade petroleum hydrocarbons. Prolonged application of chemical surfactants alters the microbial community in the environment and causes serious environmental problems [6].

Biosurfactant production large-scale industrial application is associated with increased production cost. Hence, it is an urgent need to formulate a suitable culture medium to produce biosurfactants using low-cost substrate to minimize the production cost. In recent years, efforts have been made to minimize the production of surfactants by enhancing the application and use of low-cost feed stock, cost-free feed stocks, or agro-industrial residues as substrates for the production of biosurfactants by microbial communities either in solid-state fermentation or submerged fermentation. Solid-state fermentation (SSF) has many advantages over submerged fermentation [8]. In solid-state fermentation, bacteria grow in their natural habitat and are capable of producing various enzymes and secondary metabolites that are usually produced in low amounts or not produced in submerged fermentation. In recent years, many agro-industrial wastes have been used for the production of biosurfactants in SSF by *Bacillus subtilis* [9]. Although biosurfactants have numerous advantages and various applications in industrial processes, their use is highly limited because of their high production cost and low yield. Process parameters such as the pH of the medium, incubation temperature, nutrient composition such as nitrogen, carbon, and mineral sources, and salinity are the very important factors for the optimization of production of biosurfactants. Designing experiments via a statistical approach has been used to enhance yield by optimization strategies in biotechnological process. Among the methods available, optimization of process parameters via response surface methodology (RSM) is a highly effective and widely used statistical method for experimental design, evaluating various factors and searching the optimum bioprocess conditions [10]. RSM has various advantages, including being less time-consuming, less laborious than other methods, and able to analyze a design with the minimum number of experimental runs [11]. Additionally, we can evaluate the interactive effect of all the selected variables simultaneously that are used in the experiment using RSM. RSM has been successfully employed for the production of various biomolecules, including biosurfactants [12]. A statistical approach has been widely used for the production of various biomolecules; however, the statistical approach for the production of biosurfactants is highly limited [13]. The central composite design and response surface methodology (RSM) is widely used to evaluate the relationship between one factor or more and a set of experimental variables. RSM allows evaluation of the variables and their effects on many components of the medium at various concentrations. The aim of the study was to optimize the culture medium components to enhance the production of biosurfactants.

## 2. Materials and Methods

### 2.1. Bacteria and Culture Condition

In this study, *Bacillus subtilis* was isolated from marine soil sediment in Saudi Arabia. A total of five samples were collected from the Eastern Province along the Arabian (Persian) Gulf. Each sample was serially diluted (10-fold dilution) and plated on nutrient agar, and incubated at 37 ± 2 °C for 24 h. Pure culture was obtained by plating the isolate on the same nutrient agar using the single colony isolation method. Separated pure colonies were maintained on nutrient agar slants for further use. The morphologically different bacterial isolates were labeled separately and used for biosurfactant production.

### 2.2. Biosurfactant Screening

#### 2.2.1. Blood Hemolysis Test

A total of 109 morphologically different bacterial strains were isolated from all five stations and subjected to biosurfactant screening. The biosurfactant-producing ability of the selected bacterial strain was tested using the blood hemolysis technique. Briefly, the bacterial strains were grown on blood agar plates and incubated at 37 ± 2 °C. The clear zone formation around the bacterial strain confirmed the production of biosurfactants [14].

#### 2.2.2. Oil-Spreading Technique

Morphologically different bacterial strains were inoculated into the minimal medium (20 mL) (of (g/L): CaCl_2_:0.02, MgSO_4_:0.2, K_2_HPO_4_:1, KH_2_PO_4_:1, FeCl3.6H_2_O:0.05, NH_4_NO_3_:1, pH 7.0) containing 2% diesel oil as the sole source of carbon. The culture was incubated for 24 h andcentrifuged at 10,000 rpm for 10 min. The cell-free extract was used as the sample for the oil-spreading method. The oil-spreading technique was performed in duplicate through the addition of 20 mL of double-distilled water to Petri dishes. To this, vegetable oil (100 μL) was incorporated at the surface of the water. Then, 20 μL cell-free biosurfactant solution was dropped on the surface of the oil. The formation of zone on the oil surface was measured after 30 min of incubation.

### 2.3. Characterization of Potent Surfactant-Producing Bacterial Strain

The morphological and biochemical characters of the maximum biosurfactant-producing bacterium were analyzed [15]. The 16S rDNA analysis of the potent strain was performed for molecular level identification. Briefly, the chromosomal DNA of the selected strain was extracted and purified using a DNA purification kit (Promega DNA purification kit, Madison, WI, USA). The universal forward (5′AGTTTGATCCTGGCTCAG3′) and reverse primer (5′AAGGAGGTGATCCAGCC3′) were used for gene amplification using a “Gene Amp PCR system” (Thermo Fisher Scientific, Waltham, MA, USA). The PCR master mix (25 μL) consisted of deoxynucleoside triphosphate (0.2 mM), primer (1.32 μM), DNA polymerase (0.5 U), buffer (5 μL), and 5ng DNA template. The PCR program was 94 °C for 3 min followed by 35 cycles consisting of denaturation at 94 °C for 45 s, annealing at 59 °C for 1min, and extension at 72 °C for 2 min. The amplified 16S rDNA gene was separated using 1% (*w*/*v*) agarose gel electrophoresis. The amplified gene was purified (Promega Gel Extraction Kit, Madison, WI, USA) and sequenced with ABI PRISMTM 3100 Genetic Analyzer (Applied Biosystems, St. Louis, MO, USA).

### 2.4. Inoculum

*Bacillus subtilis* strain Al-Dhabi-130 was carefully streaked on nutrient agar plates and incubated for 24 h at 37 ± 2 °C. After 18 h incubation, a loopful culture was added in 50 mL of culture medium (LB) and incubated at 37 °C for 18 h. About 200 μL broth was inoculated to 250 mL Erlenmeyer flask containing LB medium (50 mL). It was incubated at 37 °C using an orbital shaker at 150 rpm and used as inoculum for all experiments.

### 2.5. Solid-State Fermentation (SSF)

The substrates, which included rice bran, date molasses, soya meal, corn starch, and banana peel, were weighed individually. The substrate was transferred into 100 mL Erlenmeyer flask. All substrates were sterilized in an autoclave at 121 °C for 1 h to eliminate microbial spores and cooled. Then, 100 μL of 1 M KH_2_PO_4_, 250 μL 1M MgSO_4_, and 350 μL double-distilled water was added to enrich the culture medium. To this culture medium, 1.0 mL bacterial strain was inoculated, mixed carefully, and incubated at 37 °C for 48 h. Biosurfactant was evaluated at 24 h intervals, all experiments were performed in triplicate analysis, and the mean value was considered.

### 2.6. Extraction and Assay of Biosurfactants

The crude sample was used for the analysis of surfactants. The bacterial cells were removed by centrifugation (10× *g* for 10 min), and the proteins and lipids from cell-free extract were precipitated by adding concentrated hydrochloric acid (pH 2.0). Finally, a grey white pellet obtained after precipitation was isolated by centrifugation for 4 °C at 10× *g* for 10 min. It was lyophilized and used for quantification assay. Then, 50 mL chloroform–methanol mixture (2:1) was added to the sample to a final chloroform/methanol/water ratio of 2:1:0.75. Then, the mixture was centrifuged (10,000 rpm, 10 min), the organic layer was carefully collected, and the sample was evaporated to dryness under N2 at 37 °C for 30 min [16].

### 2.7. Emulsification Assay

The crude sample (0.05 mg/mL) was mixed with Tris-buffer (20 mM, pH 7.0) containing 10 mM MgSO_4_ and 0.1 mL kerosene. It was shaken vigorously using a vortex, and the tubes were kept for an hour at 32 ± 2 °C. Finally, the emulsification activity was observed by reading the OD at 540 nm against reagent blank.

### 2.8. Surface Tension Analysis

Surface tension analysis was carried out to analyze the surface activity of the synthesized biosurfactant by *B. subtilis* strain Al-Dhabi-130.A Tensiometer (VWR International, Radnor, Pennsylvania, PA, USA) was used to analyze the surface tension of the sample.

### 2.9. Screening of Solid Substrates

Substrates such as rice bran, date molasses, soya meal, corn starch, and banana peel were used for optimization studies. Based on the biosurfactant assays described in Section 2.4, date molasses showed the best biosurfactant production potential under the set experimental conditions and were thus selected for optimization. Hence, 10 g sterilized date molasses was used as the substrate for the one-variable-at-a-time approach and response surface methodology. Extraction and biosurfactant assay was carried out as described previously.

### 2.10. Proximate Analysis of Date Molasses

Date molasses was subjected to analysis of their nutrients and physical properties. Moisture, tannin, fiber, fat, total protein, sugar, pectin, and ash contents were analyzed. Total protein content of the sample was estimated as per Lowry et al. [17]. The total carbohydrate from the date molasses and other nutrients were tested based on the methods provided in the FAO guide [18].

### 2.11. Optimization of Biosurfactant Production via the One-Variable-At-A-Time Approach

The one-variable-at-a-time approach was initially used to screen the variables. The selected variables were pH (6.0–9.0), temperature (25–40 °C), glucose (5–10%), and incubation period (3–7 days). Date molasses was used as the substrate for the production of biosurfactants. It acts as the primary source of carbon and nitrogen and also helps to anchor bacterial cells.

### 2.12. Screening of Variables Influencing Biosurfactant Production via the Statistical Approach

A two-level full factorial design was used to screen biosurfactant production in solid-state fermentation (SSF). Two-level full factorial designs are a more efficient method to screen medium components than other methods. In this study, a two-level full factorial design was used to evaluate the relative significance of five variables for the production of biosurfactants by *B. subtilis*. Two levels were used (low (−) and high (+)) to screen the response (product yield) in solid-state fermentation (SSF). The factors that were selected for this study were moisture (A), temperature (B), pH (C), incubation period (D), and glucose (E).

### 2.13. Central Composite Design and Response Surface Methodology

Central composite design (CCD) was selected to evaluate the optimum level of the variables affecting biosurfactant production for *B. subtilis*. CCD consists of 20 experimental runs for the selected three variables. These three variables were selected based on the outcome of two-level full factorial experimental designs. The selected factors were moisture (X1), incubation period (X2), and glucose (X3). All three factors were evaluated at five different levels (−1.682, −1, 0, +1, and +1.682). The statistical software Design expert (version 12) (Minneapolis, MN 55413, USA) was used to design and analyze the experimental model. Two different experimental trials were performed, and the average value was considered as response Y (biosurfactant yield). Analysis of variance (ANOVA) was applied to evaluate the designed CCD model for enhanced production of biosurfactants. A *p*-value of <0.05 was considered of statistical significance. The optimum response of the model and the predicted culture conditions were tested and validated in three experimental runs, and the average was considered.

### 2.14. Infrared Analysis (FT-IR)

About 1.0 mg biosurfactant was mixed with 100 mg KBr, and the mixture was pressed. The transparent pellet (134 MPa) was formed after 2–3 min. The FT-IR spectrum of the formed pellet was analyzed ranged from 400 to 4000 wave numbers (cm^−1^).

### 2.15. Analysis of Polysaccharide Using Nuclear Magnetic Resonance Spectroscopy

^1^H nuclear magnetic spectrum was observed on a 500 MHz NMR spectrophotometer (Bruker Avance II-500 spectrometer, Germany) at room temperature (30 ± 2 °C). Approximately 1–2 mg of the purified biosurfactant was prepared, and NMR spectrum was obtained. Chemical shifts were analyzed in ppm [19].

### 2.16. MALDI-TOF Analysis of Biosurfactants

The purified biosurfactant (0.1 μL) was placed onto the anchor chip position on a MALDI plate. Then, 1 μL matrix was carefully added to the sample spot. Peptide standard was used as the external standard. The matrix was prepared by adding α-cyano-4-hydroxycinnamic acid and 2,5-dihydroxybenzoic acid. The sample spot was allowed to dry, and the spectrum was determined using a mass spectrometer with 20 kV. The data were processed using data explorer 4.4.

### 2.17. Gas Chromatography—Mass Spectophotometry (GC-MS) Analysis

The fatty acid components of biosurfactant were analyzed using a Thermo Trace GC apparatus equipped with a DB-5 column (0.25 mm × 30 m × 0.22 μm), attached with Polaris Q MS. An amount of 10 μL of sample was injected, and helium was used as a carrier gas. The oven temperature was maintained between 60 °C and 260 °C. The run time was 30 min, and the flow rate was 1 mL/min. The initial temperature (60 °C) was maintained for 2 min, and the final temperature was 260 °C. The MS of detected fatty acid methyl ester was compared with the National Institute of Standards and Technology (NIST) database.

### 2.18. High Performance of Liquid Chromatography

The biosurfactant sample was hydrolyzed with HCl (6 M) containing 0.2% phenol. It was incubated for 24 h at 110 °C in closed vials and further dried under reduced pressure. Amino acid analyses of biosurfactant were performed using a HPLC system Agilent 1100 HPLC instrument (Massy, France) equipped with C18 reverse-phase column. An amount of 10 μL of sample was injected and eluted with 65% methanol containing a13 mM trifluoroacetic acid (isocratic elution). The flow rate of the sample was maintained as 1.0 mL/min; eluted amino acids were detected at 340 nm and compared with commercial standard.

### 2.19. Degradation of Crude oil by Bacillus subtilis Strain Al-Dhabi-130

In this study, the crude oil degradation efficacy of *B. subtilis* strain Al-Dhabi-130 was evaluated in mineral liquid medium, which was composed of [g /L] 2.0 KH_2_PO_4_, 1.0 NH_4_NO_3_, 3.0 Na_2_HPO_4_, 0.7 KCl and trace metal solution (0.2 FeSO_4_, 4.0 MgSO_4_, 0.2 CaCl_2_ and 0.2 MnCl_2_). The culture medium was sterilized by autoclaving at 121 °C for 20 min and cooled. Then, 1% crude oil was filter-sterilized and supplemented as the sole source of carbon. The sterilized medium was inoculated with *B. subtilis* strain Al-Dhabi-130 to a 250 mL Erlenmeyer flask containing minimal medium. A control was also maintained without any bacterial inoculation. The flasks were incubated for 2 weeks at 30 ± 2 °C at 175 rpm using a rotary shaker. After 2 weeks, the crude oil residues in the bacterial culture were analyzed using GC-MS.

### 2.20. Desorption of Crude Oil—Polluted Environment

A roughly 2 kg soil sample was collected from the marine environment. It was air-dried at room temperature and sieved using a 2 mm size standard sieve [20]. Crude oil was mixed for 1 h with the soil sample to reach the final concentration of 500 mg kg^−1^. Further crude oil desorption from oil-contaminated soil was evaluated. About 5.0 g of soil sample artificially contaminated with crude oil was taken in a pre-sterilized Erlenmeyer flask, and 50 mL biosurfactant from *B. subtilis* strain Al-Dhabi-130 was added. The Erlenmeyer flask was kept on a shaker (150 rpm) for a week at 30 ± 2 °C. After that, the remaining crude oil from the soil sample was extracted with acetone and hydrocarbon, and its derivatives were analyzed using GC-MS analysis.

## 3. Results and Discussion

### 3.1. Biosurfactant-Producing B. subtilis

**Figure 1:**
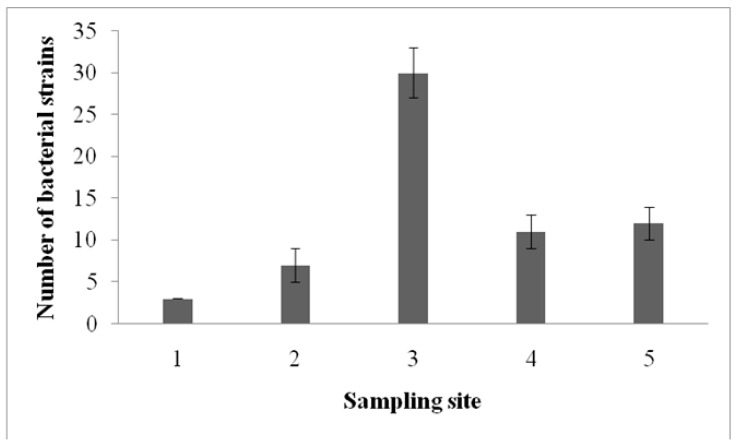
Distribution of biosurfactant-producing bacterial strains in oil-contaminated site.

A total of 63 surfactant-producing bacterial strains were isolated from the crude-oil-contaminated marine soil sediment. The population of biosurfactant-producing bacterial strains was abundant in three among the five sampling sites. These isolates were screened based on various biosurfactant screening tests. The isolates showed a positive result in the blood hemolysis and oil-spreading tests. Among the 63 isolates, 40 were Gram positive, and the remaining 23 were Gram negative. In sample site three, a maximum of 30 positive strains were isolated (Figure 1). It may be noted that this site was more polluted with crude oil than the other selected sites. In sampling site one, only three biosurfactants producing bacterial isolates were isolated.

### 3.2. Screening of Agro-Industrial Waste for the Production of Surfactants

**Figure 2:**
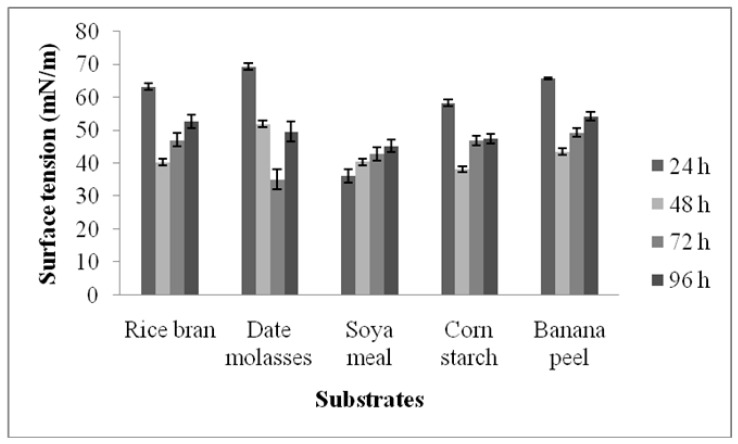
Effect of various substrates on biosurfactant production by *B. subtilis* strain Al-Dhabi-130.

Biosurfactant-producing *B. subtilis* isolated from oil-contaminated soil was subjected to screening for suitable substrates for surfactant production. The experiments on the production of biosurfactants were performed for 72 h. The selected substrates, such as rice bran, date molasses, corn starch, and banana peel, enhanced biosurfactant production after 48 h; however, soya meal stimulated more enzyme production within 24 h. In the third day of incubation, date molasses enhanced biosurfactant production significantly more than other substrates. Among the selected agro-industrial wastes, surfactant production was high in the date molasses medium. The biosurfactant yield was at maximum after 72 h, and surface tension was 35.2 ± 2.6 mN/m in date molasses substrate. After 96 h incubation, the surface tension of the sample increased, which indicated decreased biosurfactant production (Figure 2). The application of various low-cost substrates for biosurfactant production has various advantages, including a reduction in the production cost of enzymes in the industrial scale. Hence, the selection of an ideal fermentation medium is a key factor for the production of biomolecules, including biosurfactants [8]. In this study, date molasses and rice bran improved biosurfactant production compared to other tested substrates. In these substrates, the nutrient compositions vary widely. Date molasses consists of 18% moisture content, 0.1% plant tannin, 2.3% protein, 73.1% carbohydrate, and 2.1% ash content. Date molasses is a cheap renewable carbon source used for the production of biosurfactants. It is rich in sugar and is a promising substrate for biosurfactant production. Date molasses contains carbohydrates (67–70%), minerals, organic acids, and vitamins [21]. Rice bran contains 18.8% fat, 13.66% protein, 40.63% carbohydrate, 12.48% fiber, and γ-oryzanols, α-tocopherol, and γ-tocopherol [22]. These nutrients improved biosurfactant production. Recently, the low-cost substrate corn steep liquor has been used as a potential nitrogen source for the production of biosurfactants, reported by Santos et al. [23] and Luna et al. [7]. The production of biosurfactants by *B. subtilis* using date molasses as a potential novel source has been performed by Al-Bahry et al. [24] and Al-Wahaibi et al. [25].

### 3.3. Screening of Variables for Biosurfactant Production via the One-Variable-At-A-Time Approach

**Figure 3:**
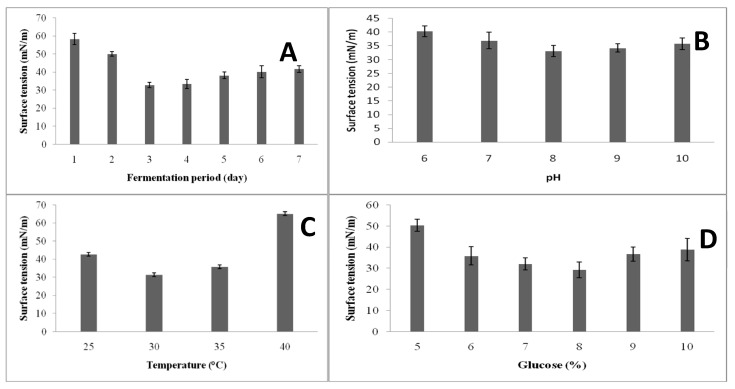
Effect of fermentation period (**A**), pH (**B**), temperature (**C**), and glucose concentration (**D**) on biosurfactant production.

The one-variable-at-a-time-approach was initially used to screen the variables. The selected variables were pH (6.0–10.0), temperature (25–40 °C), glucose (5–10%), and incubation period (1–7 days). Carbon sources are required for the production of biosurfactants, and these organisms require a very large amount of oxygen. Water-soluble substrates have been utilized for the production of surfactants more often than oil substrates. Date molasses acts as the primary source of carbon and nitrogen and also helps to anchor bacterial cells [26]. In this study, surfactant production was at its maximum after 72 h of incubation indicated low surface tension (32.9 ± 1.5 mN/m than 96 h (Figure 3A) using date molasses. Hence, in subsequent experiments, 72 h was used. Likewise, 72 h was found to be optimum for the production of surfactants in *Bacillus subtilis* BS5 [27]. In this study, the initial pH of the culture medium was adjusted as 6.0, 7.0, 8.0, 9.0, and 10.0. At pH 8.0, biosurfactant production was found to be maximum (33.1 ± 2 mN/m) compared to other tested pH values (Figure 3B). To monitor the influence of pH on biosurfactant production, the emulsifying activity was monitored continuously, and the yield was recorded. It was previously reported that in acidic pH, biosurfactant yield decreased due to the formation of precipitates. In *Pseudomonas putida* MTCC 2467, pH 8.0 was reported to be optimum for the production of biosurfactants [28], which was similar with the current study. The results show in Figure 3C that the *B. subtilis* strain could produce maximum surfactants at 30 °C (31.5 ± 2.2 mN/m). Environmental parameters such as temperature and pH significantly affect biosurfactant production in various microorganisms. The temperature optimum for biosurfactant production is similar tothat for other *Bacillus subtilis* strains [29]. In this study, water-solubilizing glucose was used (5–10%) to enhance surfactant production. At 8% glucose concentration, the emulsifying ability was found to be high, and this concentration was suitable for this organism. Up to 8% initial glucose concentration, surfactant production was not improved, and an increased surface tension measurement was obtained. At 8% glucose concentration, surface tension was 29.3 ± 3.7 mN/m (Figure 3D). In *Bacillus* sp., an initial glucose concentration of 70 g/L has been found to be optimum for the production of biosurfactants [30]. Higher glucose concentration in the culture medium may affect glucose consumption of bacteria, thus affecting the inhibition of biosynthesis of surfactants [31].

### 3.4. Optimization of Medium Components by a Two-Level Full Factorial Design

The two-level full factorial experiments enable the identification of various factors, including optimum moisture content of the substrate (60% and 90%), temperature (25 and 40 °C), pH (7.0 and 9.0), incubation period (2 and 5 days), and glucose (5 and 10). Table 1 presents the findings of the 2^5^ level full factorial experimental designs and experimental results. Results presented in this table indicate a varying surface tension from 27.2 to 60.3 mN/m. Table 2 shows the results of analysis of variance (ANOVA) of this experimental model. In this experiment, the most significant factors were moisture content, temperature, incubation period, and glucose concentration. The other selected factor, initial pH of the culture medium, also positively influenced biosurfactant production; however, the surface tension value was very revealed little impact on biosurfactant production (*p* >0.05). The designed experimental model was statistically significant: the model F-value was 5.32, and the *p*-value of the model was 0.0019. In this model, the terms B, C, D, and E were statistically significant. The R^2^ value of this model was 0.8804, and the adjusted R^2^ value was 0.878. The equation in terms of coded factors can be used to make predictions about the response for given levels of each factor.

Biosurfactant Yield (surface tension (mN/m)) = +63.85 − 1.35A + 4.40B + 4.41C + 5.48D − 3.97E + 1.60AB + 0.400AD + 0.4750AE + 6.34BC + 4.90BD − 8.03 BE + 5.41CD + 2.77DE − 1.78ABD + 5.678ABE + 0.100BDE + 7.78ABDE

Adequate precision usually measures the signal-to-noise ratio. In this model, a ratio of more than 4.0 (9.908) indicates an adequate signal. Additionally, a 2^3^ factorial design was employed to enhance biosurfactant production by evaluating various carbon sources, some ions, and various nitrogen sources. NH_4_NO_3_ was found to be suitable to enhance the production of biosurfactants; also, carbon and nitrogen ratio influenced biosurfactant yield. In *Bacillus subtilis* SPB1, the byproducts of olive mill were used as the low cost substrate, and a 2^3^ factorial design was used to screen the variables [32]. In another study, Fontes et al. [33] applied a 2^4^ full factorial design and response surface methodology to optimize biosurfactant production from *Yarrowialipolytica* IMUFRJ 50,682.

### 3.5. Response Surface Methodology

According to the central composite design, the initial moisture content of the solid substrate, incubation period, and glucose improved surfactant production. The central composite design result is tabulated in Table 3 and Table 4. In this model, biosurfactant production varied from 2 mg/g to 261 mg/substrate. The response (Y) was 261 mg/g substrate when the organism was incubated for 84 h, in the date molasses containing 75% moisture content and 10.7045% glucose. Biosurfactant production was very low when the culture was incubated less than a day based on CCD. The final equation in terms of coded factors was:

Surface tension (mN/m) = +214.73 + 14.74A + 33.12B + 25.06C + 31.00AB − 70.75AC + 36.00BC − 59.26A^2^ − 26.55B^2^ + 7.56C^2^

**Figure 4:**
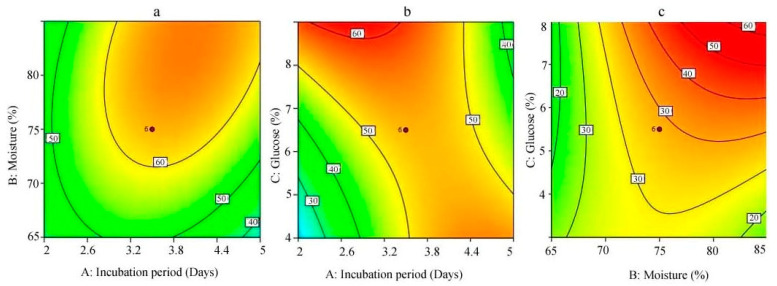
Contour plot for the surface tension property of biosurfactant produced by *B. subtilis* strain Al-Dhabi-130 at various process conditions. (**a**) Interactive effect of incubation period and moisture content, (**b**) interactive effect of incubation period and glucose, and (**c**) interactive effect of moisture and glucose.

Analysis of variance (ANOVA) was used to navigate the designed quadratic model equation, and the R^2^ value of this model was 0.9406. The F-value of this model was 14.58, which shows that this designed model was highly significant. This finding clearly indicated that experimental results of the response Y (biosurfactant yield) highly agreed with the regression value of the designed model. The adjusted R^2^ value was 0.8871, that is, the designed model could explain 88.71% of variation in the results response (Y). The lack of fit of the designed CCD model was not significant (F value = 0.891, *p*= 0.352), which shows that the designed CCD model could totally reflect the original situation. In this model, a precision of more than 4 (13.375) indicated an adequate signal. In total, the above results clearly demonstrated the good fit of the model. The 3D response surface plot revealed the interaction between incubation period, moisture, and glucose (Figure 4a–c). Among all the factors, moisture content was the most influential one, followed by glucose content and incubation period. Thus, the designed model can be used to validate and predict the effect of moisture content, incubation period, and glucose content. A response surface graph was generated from the quadratic regression equation for the production of biosurfactants. The coefficient estimate was positive for all three selected factors (incubation period, 14.74; moisture, 33.12; glucose, 25.06). An RSM plot was used to analyze the effect of interaction between relevant factors. The contour plot of the designed CCD model was elliptical and also horizontal in shape, mainly because the interactive effect of the selected variables was relatively weak. Simple sugar-like glucose or sucrose induces the production and release of surfactants from cells. CCD and RSM have been previously used to optimize various bioprocess conditions [34]. In *Acinetobacter* sp. YC-X 2, the bioprocess parameters for biosurfactant production were optimized via response surface methodology, and the synthesized biosurfactants showed stability toward various pH conditions, salinity, and high temperatures [35]. Recently, Mouafi et al. [11] optimized biosurfactant production by *Bacillus brevis*, and factors such as pH, temperature, glucose concentration, and incubation period were evaluated. The biosurfactant production ability of *Streptomyces* sp. DUPA1559 was improved by optimizing the process parameters, and the yield was 1.74 gL^−1^ after 96 h incubation [36]. In a study, Najafi et al. [13] optimized the production of biosurfactants from *Bacillus* *mycoides*, and temperature was the most significant factor for biosurfactant production.

### 3.6. Characterization of Biosurfactant

**Figure 5:**
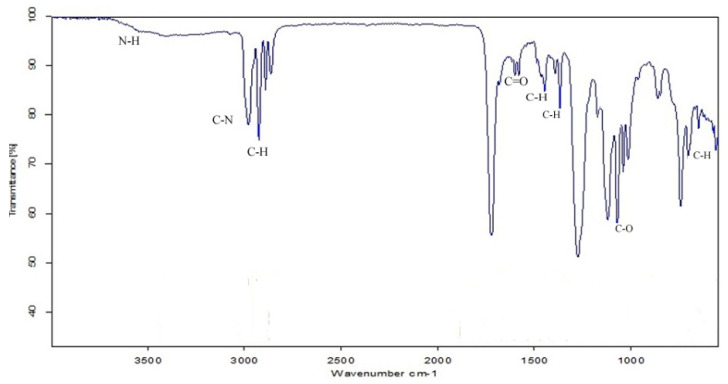
IR spectrum of biosurfactant from *Bacillus subtilis* strain Al-Dhabi-130.

IR-spectrum of biosurfactant from *B. subtilis* strain Al-Dhabi-130 showed various strongly absorbing bands, characterized by a peptide component at 1721 cm^−1^ resulting in the stretching mode of the C=O bond. At 1446 cm^−1^, C–N stretching mode combined with N–H band was observed. The C–H modes from 2972−2855 cm^−1^ and 1446−1366 cm^−1^ were analyzed (Figure 5). These findings revealed peptide-like moieties with aliphatic type. The band observed at 1721 cm^−1^ was due to lactone carbonyl absorption as observed by de Faria [37] in *Bacillus subtilis* isolate LSFM-05. In the present study, prominent absorption bands at 2924 cm^−1^, 1400 cm^−1^, and 1383 cm^−1^ indicated the presence of CH_3_ and CH_2_ (aliphatic chains and alkyl chains) in the biosurfactant. A broad peak was determined at the range between 3800 cm^−1^ and 3100 cm^−1^ indicated N–H and C–H vibrations in the biosurfactant sample.

**Figure 6:**
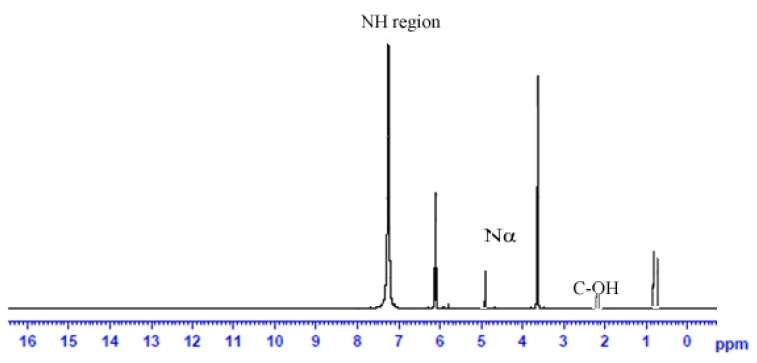
Nuclear magnetic resonance spectra of surfactant purified from *Bacillus subtilis* strain Al-Dhabi-130.

^1^HNRR analysis of the biosurfactant extracted from *B. subtilis* Al-Dhabi-130 showed that the purified biosurfactant was a lipopeptide because of the presence of aliphatic chain (CH_2_ at 0.83 to 0.75 ppm), a peptide backbone (N–Nat 8.1–7.1 ppm), and an aliphatic carbon–hydrogen bond (C–H at 4.9–5.9 ppm). The doublet signal obtained in this study between 0.83 and 0.75 ppm indicated terminal branching in the fatty acid component. The chemical shift at 5.9 ppm was consistent with proton attached to the C-3 region of the fatty acid residue, which clearly revealed that this carbon atom was attached to an amino acid residue using an ester bond (Figure 6). Tang et al. [38] reported a peak at 3.67 ppm in ^1^H NMR spectrum and confirmed the presence of methoxy group on the Asp or Glu amino residues. The ^1^H NMR analysis observed in this study revealed the presence of an ester carbonyl group at 6.1 ppm. In a study, Liu et al. [39] characterized this kind of lipopeptide produced by the bacterial strain *B. subtilis* HSO 121. The NMR spectrum confirmed the presence of a very long aliphatic chain and amide-NH group peptide backbone. Analysis of biosurfactant with ^1^H NMR and FTIR analysis suggested the isolated biosurfactant was lipopeptide. Biosynthesis of various isoforms of biosurfactants by bacteria from the genus *Bacillus* can be mainly attributed to the group of conserved genes typically responsible for these enzymes by non-ribosomal synthesis. The structural changes in the biosurfactants add advantages to survive under drastic environments.

**Figure 7:**
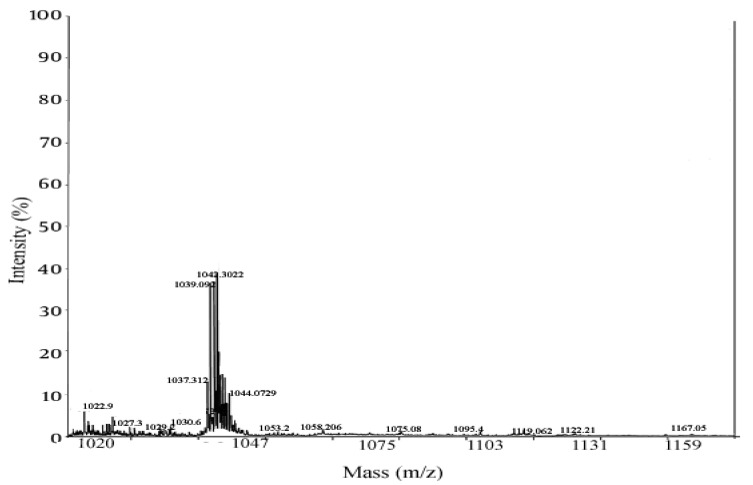
MALDI-TOF analysis of biosurfactants isolated from *Bacillus subtilis* strain Al-Dhabi-130.

**Figure 8:**
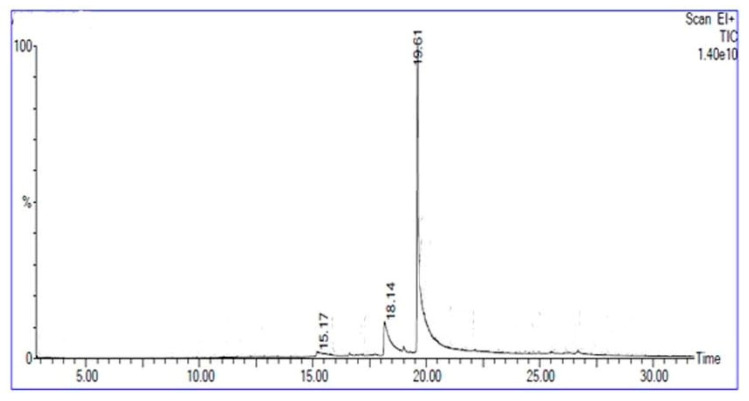
Gas chromatography profile of fatty acid methyl ester showing a major peak with retention time of 19.61 min.

The biosurfactant produced by *Bacillus subtilis* strain Al-Dhabi-130 was analyzed using MALDI-TOF, and the result was presented in Figure 7. The mass spectra showed a well resolved peak between 1030 and 1060 m/z. The biosurfactant determined in MALDI showed peaks with the m/values, 1030, 1044.07, and 1058.208, and these values were associated with heptapeptide moiety. In a study, Pecci et al. [40] purified and characterized surfactin produced by the bacterial strain, *Bacillus licheniformis* V9T14. Qiao and Shao [41] characterized lipoamino surfactant (proline lipid) from the marine bacterial strain *Alcanivorax dieselolei* strain B-5. Recently, Deng et al. [42] characterized a cyclic lipopeptide surfactant isolated and structure was elucidated from the bacterial strain *Achromobacter* sp. strain HZ01. The lipopeptide determined in the study composed of a lactone ring with four amino acid residues (Asp, Ser, Gly, and Tyr) and the ring is also linked with other amino acids by amide bond. GC-MS analysis of the hydrolyzed solvent fraction showed the presence of single major peak at the retention time of 19.62 min (Figure 8). The fragmentation profile of the ion of m/z 1042 shows the loss of a 143 Da residue. The m/s of 143 Da revealed the presence of glutamic acid residue as the methyl esterified amino acid.

**Figure 9:**
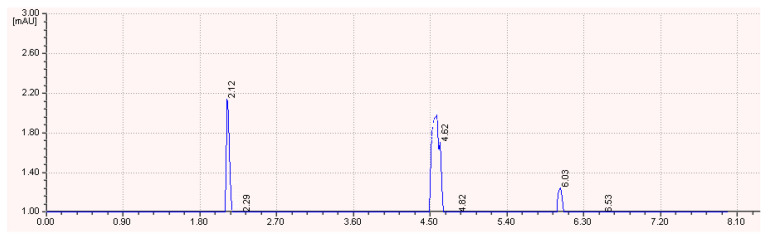
HPLC chromatogram of hydrolyzed biosurfactant from *B. subtilis* strain Al-Dhabi-130.

The HPLC analysis of the biosurfactant hydrolyzed fractions showed the amino acid peak at 2.12 min, 2.29 min, 4.62 min, 4.82 min, 6.03 min, and 6.53 min, respectively. These retention times were similar with the standard amino acids Thr-Tyr-Val-Ser-Tyr-Thr (Figure 9). The present finding revealed that the purified surfactant from the *B. subtilis* strain Al-Dhabi-130 has methylated glutamic acid moiety. The lipopeptide determined in the study composed of a lactone ring with amino acid residues and the ring is also linked with other amino acids by amide bond. The dissociation profile clearly revealed that the identified lipopeptide was a six-residue peptide (Thr-Tyr-Val-Ser-Tyr-Thr) with a lactone bond and linked with fatty acid moiety. Based on these characterization studies, the biosurfactant structure was predicted, and the structure was proposed as below:

C_14_H_26_O^−^H^+^-NHC_3_H_5_OHCOOH-NHC_8_H_7_OHCO-NHC_4_H_8_CO-NHC_2_H_3_OHCO-NHC_8_H_7_OHCO-NHC_3_H_5_OHCOOH

### 3.7. Biodegradation of Crude Oil by Bacillus subtilis Strain Al-Dhabi-130

In this study, change in the components of crude oil was observed in GC-MS analysis. The GC-MS analysis of the crude oil in the experimental sample indicated removal of various aromatic hydrocarbon and n-alkanes. It clearly indicated that the selected bacterial strain utilized n-alkanes and various aromatic hydrocarbons and their components from crude oil. The biodegradation efficiency of this bacterial strain was calculated to be >95% after 14 days of incubation. The selected bacterial strain degraded low molecular weight compounds (between C9 and C14) and also high molecular weight components (between C15 and C20). The present finding demonstrated that the selected bacterial strain has the capacity to degrade crude oil. The biodegradation process was mainly due to the secretion of biosurfactants by the strain. The involvement of biosurfactants in the biodegradation of crude oil has been reported previously. *Bacillus subtilis* effectively produced biosurfactants, rhamnolipids, and degraded crude oil hydrocarbon [43]. In another study, the synthesis potential of biosurfactants and lipopeptides by *P. aeruginosa* and degradation efficacy of crude oil hydrocarbons was reported by Parthipan et al. [44]. These studies clearly revealed the efficacy of biosurfactants in crude oil degradation. Baoune et al. [45] characterized *Streptomyces* spp. With >98% removing the efficacy of petroleum hydrocarbons. Despite various reports on the biodegradation potential of hydrocarbons, however, degradation of bacterial strains of both aromatic and aliphatic hydrocarbons is scarce [46]. The isolated bacterial strain, *B. pumilus*, could degrade dibenzothiophene and various metabolites [47]. Catechol 1,2 dioxygenase (C12D) synthesized by *B. pumilus* MVSV3 was found to effectively degrade hydrocarbons [48]. These analyses clearly revealed the involvement of biosurfactants and other gene products in hydrocarbon degradation.

### 3.8. Desorption of Crude Oil by Surfactants

The crude biosurfactant produced by *Bacillus subtilis* strain Al-Dhabi-130 desorbed 89% of the applied crude oil from the soil sample in this study. The experiment clearly revealed that supplementation of biosurfactants to soil sediments enhances the removal of crude oil. Crude oil generally binds to soil sediment and is very difficult to remove due to its strong sorption capacity to sediment and its hydrophobicity. However, biosurfactants synthesized by bacterial isolates in crude-oil-contaminated soil sediments can enhance hydrocarbon emulsification, resulting in increasing their solubility toward water molecules, increasing the oily substance displacement and decreasing surface tension [49]. According to Iwai et al. [50] and Lee et al. [51], not only is biosurfactant involved in the degradation of crude oil, but various genes and their metabolic products are involved in the bioremediation of hydrocarbons in the marine environment. Desorption experiments revealed that the crude biosurfactants produced by *Bacillus subtilis* effectively desorbed crude oil from the marine soil. Members of the bacteria from *Rhodospirillaceae, Rhodobacteraceae, Oceanospirillaceae, Halomonadaceae, Shewanellaceae*, and *Pseudomonadaceae* have the ability to produce biosurfactants and increased biodegradation efficiency [52,53]. Crude oil is a highly complex mixture of various aromatic and aliphatic hydrocarbons with very low bioavailability to the microorganisms, thus inhibiting uptake of the carbon source for their growth and metabolism. Additionally, the presence of crude oil in the soil causes various impacts to the environment and organisms. However, some bacteria termed as “Hydrocarbonoclastic bacteria” create bioavailability for the microorganisms. The bacteria from the genera *Marinobacter*, *Alcanivorax*, *Thalassolituus*, *Oleispira*, and *Cycloclasticus* and associated genera contribute more than 90% of the total microbial population in oil spill areas [54]. In recent years, application of biosurfactants in treating oil-contaminated soil has increased. Indigenous microbes associated with the contaminated site are a key source for degrading hydrocarbon from contaminated soils. However, crude or partially purified biosurfactants may be treated with oil-contaminated soil to enhance the bioavailability of hydrocarbons to the environment, because the soil from the marine environment is not fertile enough compared to agricultural soil. Recently, Obi et al. [55] isolated various oil-sludge-degrading bacteria from the soil and reported that bacteria from the genera *Pseudomonas*, *Stenotrophmonas*, *Bacillus*, *Bordetella*, *Brucella*, *Ochrobactrum*, *Achromobacter*, *Mycobacterium*, *Advenella*, *Mesorhizobium*, *Pusillimonas, Klebsiella*, and *Raoultella* showed hydrocarbon-degrading efficacy. Biosurfactants were efficiently produced by the indigenous bacteria using low-cost agro-industrial wastes, which highly reduces the production cost of biosurfactants. Biosurfactants produced by indigenous strains are not highly competitive compared to chemical surfactants from an economy point of view. Hence, large-scale production of biosurfactants using cheap substrates is required to minimize production cost using indigenous bacteria [56].

## 4. Conclusions

Biosurfactants are less toxic, ecofriendly, biodegradable, and active under extreme pH and temperature. In this study, 63 biosurfactant-producing crude-oil-degrading bacteria were screened from marine sediments. A potent biosurfactant-producing indigenous bacterium was selected based on preliminary screening. It utilized date molasses as a cheap substrate for the production of bacterial biosurfactants. Biosurfactant yield improved after 72 h, pH 8.0, 30 °C, 8% glucose concentration, and surface tension was 35.2 ± 2.6 mN/m, 33.1 ± 2 mN/m, 31.5 ± 2.2 mN/m, and 29.3 ± 3.7 mN/m, respectively. The yield was improved by optimizing medium components via a statistical approach. The selected *Bacillus subtilis* strain Al-Dhabi-130 utilized crude oil as its sole carbon source, effectively decreasing the amount of residual crude oil. In desorption experiments, the crude biosurfactantsproduced under optimized condition desorbed 89% of applied crude oil in soil. Overall, the biosurfactant-producing bacterium screened in this study showed decreased surface tension and effectively degraded crude oil.

## Figures and Tables

**Table 1:** Screening of factors for the production of biosurfactant using 2^5^ full factorial experimental design.

Run	A:pH	B:Moisture	C:Glucose	D:Temperature	E:Incubation Period	Surface Tension (mN/m)
1	7	60	10	40	2	49.4
2	9	90	5	25	5	58.4
3	9	90	10	25	2	30.6
4	7	60	10	40	5	29.8
5	9	90	5	40	2	38.4
6	9	90	10	40	5	28.3
7	9	90	5	40	5	45.2
8	7	60	5	25	5	31.2
9	9	60	10	40	5	48.3
10	7	60	5	25	2	41.7
11	9	60	10	40	2	39.5
12	7	90	10	25	5	46.3
13	7	60	5	40	5	40.1
14	7	60	10	25	5	51.5
15	7	90	5	25	5	59.2
16	9	60	5	40	2	46.9
17	9	90	10	40	2	39.8
18	9	60	5	25	2	46.5
19	9	90	10	25	5	50.8
20	7	90	10	40	2	27.2
21	7	90	5	40	5	50.2
22	7	90	10	40	5	32.6
23	9	60	5	25	5	47.3
24	9	60	10	25	2	51.7
25	7	60	10	25	2	42.8
26	7	90	5	25	2	43.1
27	7	90	10	25	2	37.5
28	7	60	5	40	2	60.3
29	9	60	5	40	5	50.3
30	7	90	5	40	2	30.9
31	9	60	10	25	5	48.1
32	9	90	5	25	2	44.7

**Table 2:** Analysis of variance on biosurfactant production by *B. subtilis* strain Al-Dhabi-130 in two-level full factorial design.

Source	Sum of Squares	df	Mean Square	F-Value	*p*-Value
Model	12,016.53	18	667.59	5.32	0.0019
A: pH	58.32	1	58.32	0.4645	0.5075
B: Moisture	619.52	1	619.52	4.93	0.0447
C: Glucose	1315.85	1	1315.85	10.48	0.0065
D: Temperature	959.22	1	959.22	7.64	0.0161
E: Incubation period	505.62	1	605.62	5.03	0.0490
AB	81.92	1	81.92	0.6525	0.4338
AD	5.12	1	5.12	0.0408	0.8431
AE	7.22	1	7.22	0.0575	0.8142
BC	1285.24	1	1285.24	10.24	0.0070
BD	768.32	1	768.32	6.12	0.0279
BE	2060.82	1	2060.82	16.41	0.0014
CD	937.44	1	937.44	7.47	0.0171
DE	246.42	1	246.42	1.96	0.1846
ABD	100.82	1	100.82	0.8030	0.3865
ABE	1067.22	1	1067.22	8.50	0.0120
ADE	62.72	1	62.72	0.4996	0.4922
BDE	0.3200	1	0.3200	0.0025	0.9605
ABDE	1934.42	1	1934.42	15.41	0.0017
Residual	1632.18	13	125.55		
Cor Total	13,648.72	31			

**Table 3:** Central composite design for the selected three variables for the production of biosurfactants in solid-state fermentation.

Run	A:Incubation Period	B:Moisture	C:Glucose	Surface Tension
	Days	%	%	(mN/m)
1	6.02269	75	6.5	51.2
2	3.5	91.8179	6.5	49.5
3	3.5	75	6.5	30.6
4	3.5	75	6.5	30.9
5	3.5	75	6.5	25.9
6	0.977311	75	6.5	56.7
7	3.5	75	6.5	20.4
8	2	85	4	54.9
9	3.5	75	10.7045	18.3
10	3.5	75	6.5	22.5
11	5	85	4	21.8
12	3.5	75	6.5	24.8
13	2	85	9	19.1
14	3.5	75	2.29552	42.8
15	2	65	9	28.7
16	2	65	4	50.3
17	5	65	9	55.4
18	5	65	4	29.4
19	3.5	58.1821	6.5	51.0
20	5	85	9	20.3

**Table 4:** Analysis of variance for the designed central composite design for biosurfactant production.

Source	Sum of Squares	df	Mean Square	F-Value	*p*-Value
Model	1.449 × 10^5^	9	16,098.34	17.58	<0.0001
A: Incubation period	2966.30	1	2966.30	3.24	0.1021
B: Moisture	14,980.66	1	14,980.66	16.36	0.0023
C: Glucose	8573.47	1	8573.47	9.36	0.0120
AB	7688.00	1	7688.00	8.40	0.0159
AC	40,044.50	1	40,044.50	43.73	<0.0001
BC	10,368.00	1	10,368.00	11.32	0.0072
A^2^	50,603.86	1	50,603.86	55.26	<0.0001
B^2^	10,161.17	1	10,161.17	11.10	0.0076
C^2^	824.64	1	824.64	0.9005	0.3650
Residual	9157.11	10	915.71		
Lack of Fit	4100	5	820	0.899	0.352
Pure Error	646.83	5	129.37		
Cor Total	1.540 × 10^5^	19			
